# Potential probiotic (*L. plantarum, L. pentosus, P. pentosaceus, W. cibaria* and *L. rhamnosus*) alleviates memory impairment in lipopolysaccharide‐induced cognitive dysfunction and neurotoxicity in mice brain via protection against neuroinflammation and neurodegeneration

**DOI:** 10.1002/alz.089920

**Published:** 2025-01-09

**Authors:** Abiola Oluwatosin Obisesan, Samuel O Dodo, Funmilayo Racheal Adeniyi, Juliet Nnenda Olayinka, Olusegun Adebayo Adeoluwa, Adedamola O Fafure

**Affiliations:** ^1^ Afe‐Babalola University, Ado‐Ekiti, Ekiti State Nigeria; ^2^ Afe Babalola University, Ado‐Ekiti (ABUAD), Ado‐Ekiti, Ekiti state Nigeria; ^3^ Afe Babalola University, Ado Ekiti, Ekiti Nigeria; ^4^ Afe Babalola University, Ado Ekiti, Ado Ekiti, EKITI STATE Nigeria; ^5^ AFE BABALOLA UNIVERSITY, ADO‐EKITI Nigeria; ^6^ Afe‐Babalola University, Ado‐Ekiti, Ado‐ekiti, Ekiti State Nigeria; ^7^ Afe Babalola University, Ado Ekiti, Ekiti State Nigeria

## Abstract

**Background:**

The impact of probiotics as gut and immunological modulator in restoring gut microbial balance and immune cells expression have generated much attention in the health sector. Its inhibitory effect on bacterial translocation and associated neural inflammatory processes has been reported. However, there is scarcity of data on its neuroprotective impact against neuroinflammation‐associated neurodegeneration and memory impairment. Therefore, we tested the neuroprotective capabilities of the probiotic strains against lipopolysaccharide (LPS)‐induced neurotoxicity and memory impairment in mice.

**Method:**

Forty eight mice (n = 12) weighing 25 ‐ 30 g were randomly classified into four groups consisting of probiotic strains (10^7^ CFU/ml, orally), donepezil (3 mg/kg, intraperitoneally (i.p.)), normal saline (10 ml/kg, orally), and LPS (0.25 mg/kg i.p) group. LPS was injected 60 min after treatments for seven days. Novel object recognition (NORT) and Y‐maze tests were performed 24 h following the last treatment to assess cognitive functions. Dendritic spine of the dentate gyrus was stained with Golgi cox. Immunohistochemistry detected brain‐derived neurotrophic factor (BDNF) expression as well as Iba 1 and amyloid beta activation in the hippocampus and prefrontal cortex, while enzyme‐linked immunosorbent assay was used to measure neuroinflammation (tumor necrosis factor‐α (TNF‐α), interleukin‐1 beta (IL‐1β), interleukin‐12 (IL‐12), and interferon gamma (IFN‐γ)) and oxidative stress biomarkers (glutathione (GSH) and malondialdehyde (MDA)).

**Result:**

Probiotics reduced elevation of pro‐inflammatory cytokines, MDA levels and enhanced GSH levels in the brain. Probiotic strains enhanced BDNF expressions, reduced microglia and amyloid beta activation in the hippocampus and prefrontal cortex of LPS‐induced mice.

**Conclusion:**

These finding show that probiotic strains may possess neuroprotective effect against LPS‐induced cognitive impairment and neurodegeneration via antioxidant and anti‐inflammatory mechanisms.

